# Intimal aortic sarcoma mimicking ruptured thoracoabdominal type IV aneurysm. a rare case report and review of the literature

**DOI:** 10.1186/1749-8090-6-162

**Published:** 2011-12-11

**Authors:** Panagiotis Dedeilias, Efstratios Koletsis, Ioannis Nenekidis, Achilles Chatziioannou, Pantelis Tsipas, Konstantina Dimaka, Vania Anagnostakou, Efstratios Apostolakis

**Affiliations:** 1 1st Cardiac Surgery Department, Evaggelismos Hospital, Athens, Greece; 2 Cardiothoracic Surgery Department, University Hospital of Patras, Rio, Greece; 3 1st Radiology Department, Areteion University Hospital, Athens, Greece; 4 Radiology Department, Evaggelismos Hospital, Athens, Greece

**Keywords:** angiosarcoma, intimal aortic sarcoma, thoracoabdominal aneurysm, chemotherapy

## Abstract

Primary intimal aortic sarcoma represents a very rare and highly lethal medical entity. Diagnosis is made either by embolic events caused by the tumor or by surrounding tissue symptoms such as pain. Herein we report an extremely rare case of a 51-year-old man previously operated for ascending aortic aneurysm, who presented with clinical and radiological findings suggestive of a ruptured thoracoabdominal type IV aneurysm. The patient underwent radical resection of the aorta and surrounding tissue with placement of a composite 4-branched graft. The diagnosis was made by frozen section and regular histopathologic examination of the specimen and the patient received adjuvant chemotherapy. Nine months after surgery the patient is still alive and has no signs of recurrence. We review the literature and discuss the option of postoperative chemotherapy.

## Background

Primary aortic malignant tumors are rare and have poor prognosis. The most frequent site of origin is the thoracic part of the aorta and the most common growth pattern is intimal sarcoma [1], which usually presents with embolic events, renovascular hypertension or back pain. We report an extremely rare case of an intimal sarcoma that presented as a ruptured thoracoabdominal type IV aneurysm, which was successfully operated on. We also review the literature and discuss the diagnosis and treatment options.

## Case presentation

A 51-year-old man was transferred to Evaggelismos Hospital, Athens, Greece, with recent onset of sharp, acute and persistent back pain. His medical history was significant for an ascending aortic aneurysm sized 5.5 cm, with concomitant significant aortic insufficiency. The patient underwent composite valve-graft replacement and implantation of the coronaries (Bentall operation) one year prior to the current admission.

**Figure 1:**
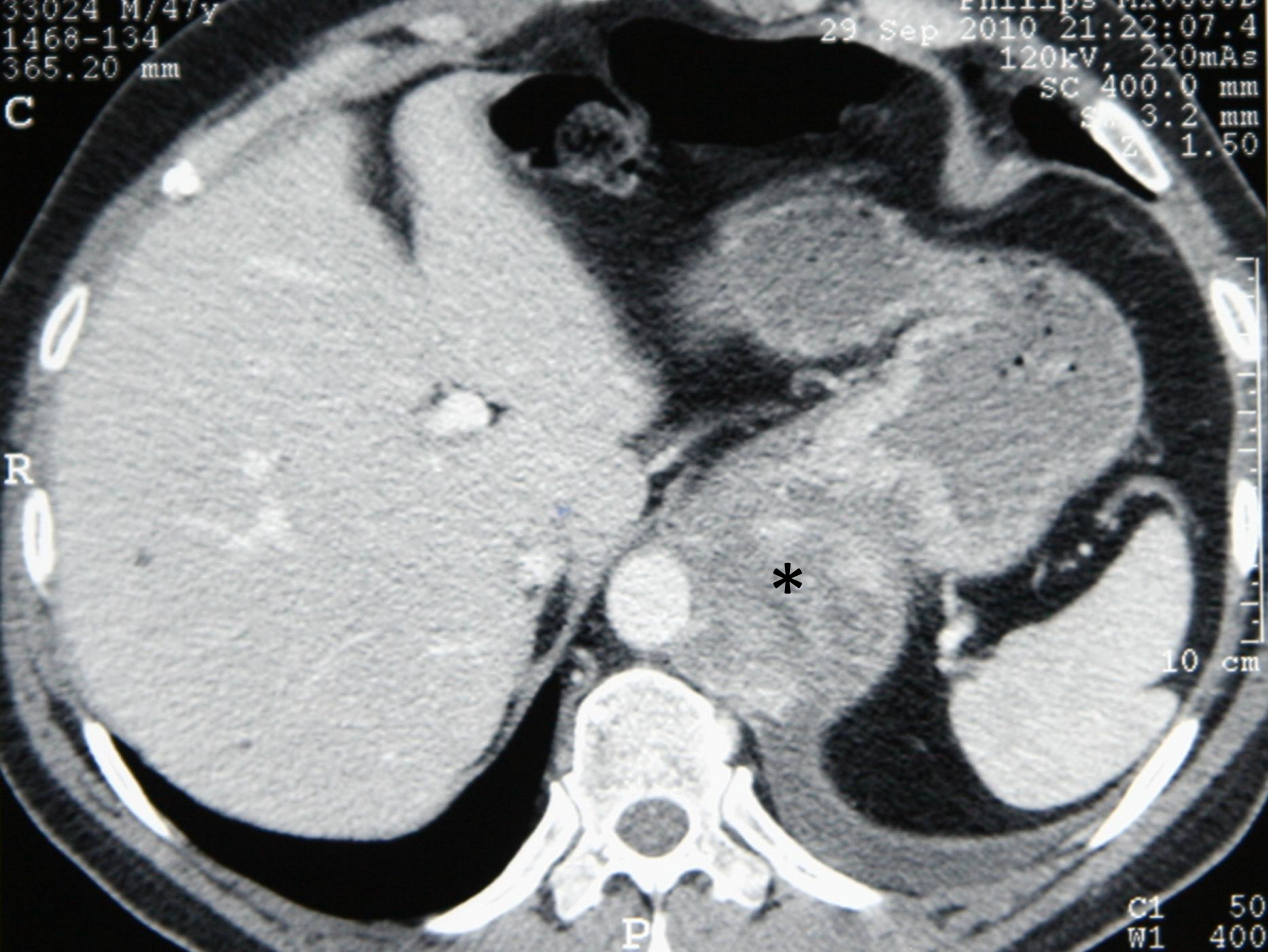
**Preoperative contrast-enhanced CT revealing the thoracoabdominal sarcoma mimicking aortic rupture**. Notice the enhancement outside the aorta (asterisk).

**Figure 2:**
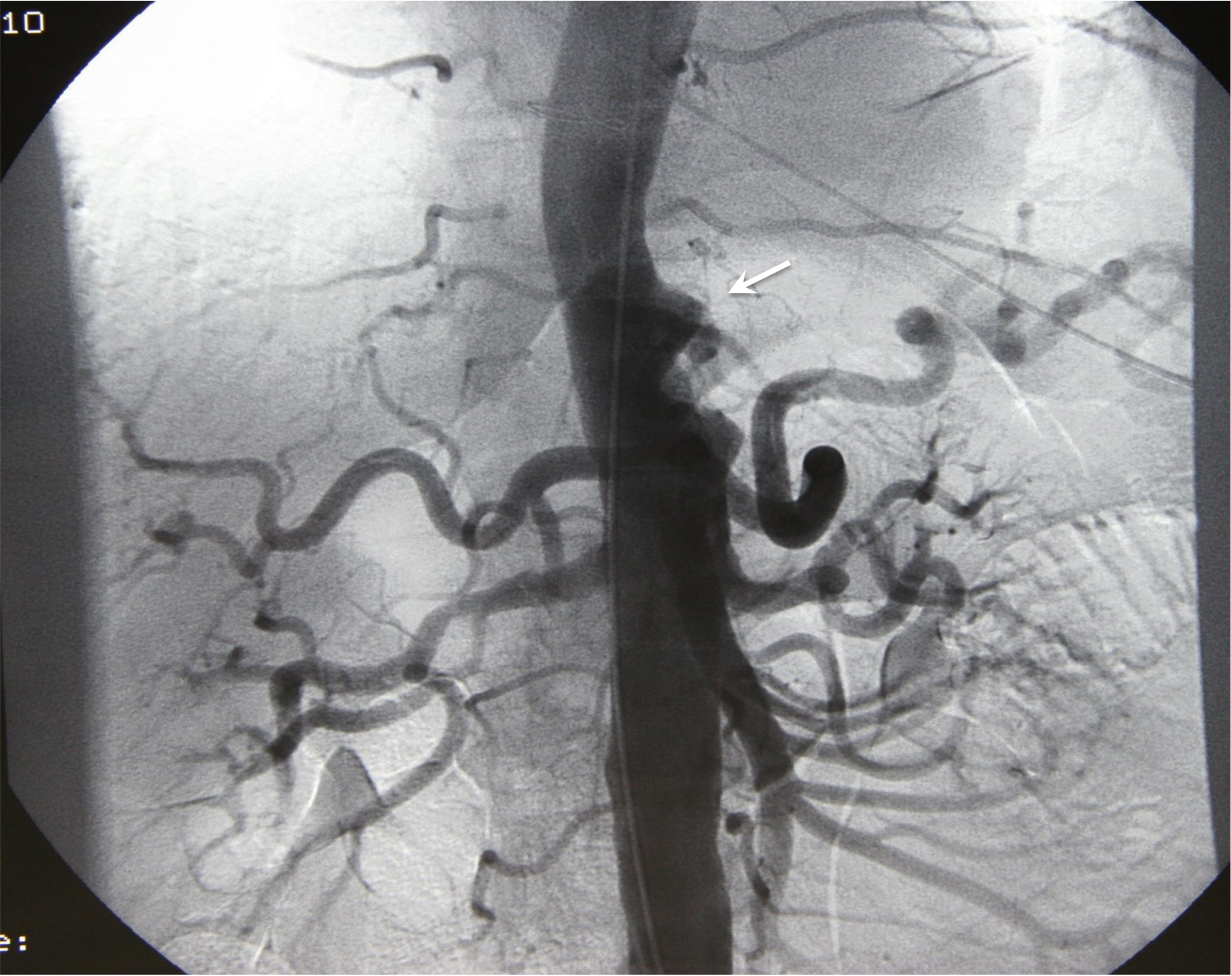
**Preoperative angiography showing disruption of the aortic wall of a thoracoabdominal type IV aortic aneurysm (white arrow)**.

Computed tomography (CT) of the chest and abdomen with contrast enhancement showed typical findings of a ruptured thoracoabdominal type IV aortic aneurysm (Figure 1). An enhanced extraluminal formation of the aorta was present, along with left-sided pleural effusion. The remaining part of the aorta was normal in size and typical postoperative findings of the previously operated side were recognized. Emergency angiography (Figure 2) confirmed the CT findings and the patient was transferred to the operating room. The patient was intubated with a double-lumen endotracheal tube and was positioned in the right lateral decubitus position. A cerebrospinal fluid (CSF) drainage catheter was placed to enhance spinal cord protection and somato-evoked potentials were monitored during rewarming. After half-dose of heparin, the left common femoral artery and vein were exposed. A 30F long cannula was inserted in the femoral vein and advanced to the right atrium. The femoral artery was cannulated with the new 18F Edwards cannula, which is longer in size and was easily advanced at the level of the iliac artery in order to achieve higher circulation flow. A left thoracoabdominal incision at the level of the sixth intercostal space was performed with additional resection of the sixth rib. At the same time, under full heparinization, the patient was put on cardiopulmonary bypass (CPB) and cooled until nasopharyngeal temperature of 15°C and bladder temperature of 16°C were reached. During the cooling period electroencephalographic silence was achieved, and 7 mg/kg of prednisolone and 10-15 mg/kg of thiopental were administered just before the aortic incision.

**Figure 3:**
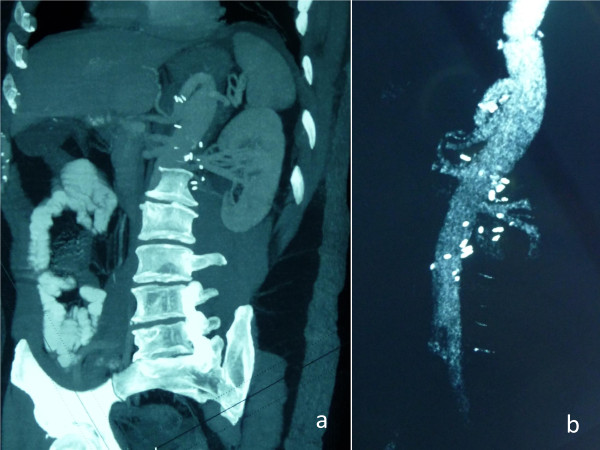
**Postoperative reconstructive spiral CT angiography demonstrating the multibranched graft placement**.

During the operation, a formation resembling a large pseudoaneurysm surrounding the lower part of the thoracic aorta was observed. With careful extraperitoneal approach the abdominal part of the aorta was mobilized. The "pseudoaneurysm" was formatted by hard granular tissue which was suspicious of a malignant formation. The celiac, upper mesenteric and both renal arteries were mobilized. Under circulatory arrest, the thoracic aorta was divided and the lower part of the thoracic aorta was excised en-bloc with the abdominal part. The abdominal branches were supplied with cold blood/normal saline solution delivered via balloon retrograde cardioplegia catheters. After the completion of the proximal anastomosis in an open fashion, the distal part of the aorta below the renal arteries was occluded. Another cross clamp was placed at the multibranched Vascutek graft (Figure 3), allowing one of the branches to de-air the upper part of the thoracic aorta, to commence CPB and start the rewarming of the patient. The total circulatory arrest time did not exceed 15 minutes. The intercostals, lumbar, visceral and renal arteries were anastomosed. After full rewarming and regaining of sinus rhythm the patient was disconnected from CPB. He was extubated 10 hours after the operation and his postoperative course was uncomplicated.

Frozen section analysis of the specimen diagnosed an angiosarcoma resected within clear margins. The immunohistochemical staining was positive for smooth muscle actin and vimentin, and negative for smooth muscle myocin, factor VIII, CD31, CD34, D2-40, keratin and symaptophysin. Approximately 50% of the malignant cells were stained by ki-67 (marker MIB-1), which confirmed that the tumor was an intimal aortic sarcoma.

One month after his discharge, the patient underwent chemotherapy with the combination treatment of ifosfamide and epirubicin. Nine months later, follow-up with CT and positron emission tomography (PET) scan has detected no signs of recurrence.

## Discussion

Primary malignant aortic tumors present very rarely. They are usually located at the descending thoracic aorta [1]. The male/female ratio is 9:5 and the mean age of the reported cases is 59.5 years [1]. There are two types of aortic sarcoma; intimal and mural [2]. The intimal type often forms intraluminal polyps or extends along the lumen, causing peripheral emboli or aortic obstruction. The mural type originates from the media or adventitia and usually extends extramurally to paraaortic tissues and lymph nodes [2-4].

Diagnosis of primary aortic tumors is difficult because of the rarity and diverse clinical manifestation of the disease [5]. Aortic sarcomas usually present with clinical signs related to embolization or nonspecific symptoms such as weight loss, fever and anorexia [6]. When the common iliac arteries are involved, the symptoms can be those of ischemia of the lower extremities or decreased peripheral pulses. If the renal arteries are involved, severe hypertension may occur. Gastrointestinal ischemia can be a result of celiac, superior or inferior mesenteric arteries involvement. Computed Tomography (CT) and Magnetic Resonance Imaging (MRI) are both useful in the differential diagnosis, with some authors advocating the superiority of MRI in detecting arterial wall sarcomas [6]. Histologic examination of the specimen is mandatory and confirms the diagnosis, with intraoperative frozen section analysis assuring adequate resection within clear margins [7].

Our case regards a primary intimal aortic sarcoma, which appears to be only the second one reported in the literature where the diagnosis was completely misled preoperatively and the patient was considered to have thoracoabdominal type IV aortic rupture [8]. In the case reported by Tanaka et al [8] there was indeed an aortic rupture caused by an intimal sarcoma, although the sarcoma was not suspected and was only diagnosed postoperatively, thus limiting the survival of their patient. In contrast, in our case preoperative imaging was misguiding and the suspicion of an underlying tumor was raised intraoperatively, frozen section biopsy was used and a radical excision of the tumor was achieved.

Regarding our patient's preoperative imaging studies, CT revealed an inhomogeneous mildly lobulated mass adjacent to the distal third of the descending aorta, extending caudally up to the level of the superior mesenteric artery. Centrally the contour of the aorta was not distorted, but more distally at the level of the diaphragm there was discontinuity of the aortic wall and the anterolateral margins of the aorta were lost. The lesion showed heterogeneous peripheral and central enhancement. A sufficient fluid collection was also present in the left hemi thorax. Taking into consideration the clinical manifestations, the patient was diagnosed with a contained ruptured aneurysm of the thoracoabdominal aorta. Angiography was also performed and showed moderate narrowing of the contour of the descending thoracic and central abdominal aorta just above the celiac artery, with an ulcer-like projection that was interpreted as a penetrating ulcer.

Prognosis for primary aortic tumors is very poor. The average survival rate is 15.6 months after diagnosis, with the mean survival being 9.8 months for intimal sarcoma [9]. Alexander et al have reported 10 cases in the English literature of primary arterial tumors arising at the anastomotic site of prosthetic woven Dacron graft implantation, though only 7 of them were fully described. The outcome of these cases was relatively poor with 4/7 dying perioperatively and the remaining ones within 10 weeks to 11 months due to metastases or recurrence [10]. They also correlate the presence of the Dacron "foreign body" with the malignant formation. They mention that the mdm-2/p53 pathway has been cited as the possible mechanism for pathogenesis of intimal sarcoma [11,12]. Thalhmeier showed 20%of tumor cells to be positive for this marker and has implicated a p53 pathway for this malignancy [13].

Primary aortic sarcomas are rarely diagnosed at an early stage due to the diversity of symptoms and the rare incidence of appearance. Since the only potentially curative modality remains radical surgical resection, this treatment option should be offered when the tumor is operable. Chemotherapy should be utilized in embolic, metastatic or nonresectable situations [14]. A combination chemotherapy regimen composed of anthracycline and an alkylating agent may offer a response rate of 20%, however the value of this regimen in the adjuvant setting for intimal aortic sarcoma is unclear [15-17]. Our patient underwent a two-drug chemotherapy treatment (ifosfamide and epirubicin) postoperatively and remains free of recurrence on follow-up MRI and PET scan studies. This could be an indication that radical excision followed by aggressive chemotherapy might be a good treatment option for this rare and highly lethal entity.

## Conclusions

Intimal aortic sarcoma is a rare medical entity with high mortality rates. It is often presented with nonspecific symptoms to the disease that can misguide the physician. A previous implantation of a woven Dacron graft might be related to a possible sarcoma formation. A high level of suspicion is required in order to reach the correct diagnosis, which is especially important for achieving a radical resection intraoperatively. The survival of the patients seems to be further improved by administration of adjuvant combination chemotherapy of two agents.

## Consent

Written informed consent was obtained from the patient for publication of this Case report and any accompanying images. A copy of the written consent is available for review by the Editor-in-Chief of this journal.

## Competing interests

The authors declare that they have no competing interests.

## Authors' contributions

All authors 1) have made substantial contributions to conception and design, or acquisition of data, or analysis and interpretation of data; 2) have been involved in drafting the manuscript or revising it critically for important intellectual content; and 3) have given final approval of the version to be published. Each author has participated sufficiently in the work to take public responsibility for appropriate portions of the content.
